# Rad5 and Its Human Homologs, HLTF and SHPRH, Are Novel Interactors of Mismatch Repair

**DOI:** 10.3389/fcell.2022.843121

**Published:** 2022-06-16

**Authors:** Anna K. Miller, Guogen Mao, Breanna G. Knicely, Hannah G. Daniels, Christine Rahal, Christopher D. Putnam, Richard D. Kolodner, Eva M. Goellner

**Affiliations:** ^1^ College of Medicine Department of Toxicology and Cancer Biology, University of Kentucky, Lexington, KY, United States; ^2^ Ludiwg Institute for Cancer Research San Diego, San Diego, CA, United States; ^3^ Department of Medicine, University of California San Diego, San Diego, CA, United States; ^4^ Moores-UCSD Cancer Center, San Diego, CA, United States; ^5^ Institute of Genomic Medicine, San Diego, CA, United States; ^6^ Department of Cellular and Molecular Medicine, University of California San Diego, San Diego, CA, United States; ^7^ Markey Cancer Center, University of Kentucky, Lexington, KY, United States

**Keywords:** mismatch repair (MMR), rad5, SHPRH, HLTF, alkylating agent MNNG, binding motif

## Abstract

DNA mismatch repair (MMR) repairs replication errors, and MMR defects play a role in both inherited cancer predisposition syndromes and in sporadic cancers. MMR also recognizes mispairs caused by environmental and chemotherapeutic agents; however, in these cases mispair recognition leads to apoptosis and not repair. Although mutation avoidance by MMR is fairly well understood, MMR-associated proteins are still being identified. We performed a bioinformatic analysis that implicated *Saccharomyces cerevisiae* Rad5 as a candidate for interacting with the MMR proteins Msh2 and Mlh1. Rad5 is a DNA helicase and E3 ubiquitin ligase involved in post-replicative repair and damage tolerance. We confirmed both interactions and found that the Mlh1 interaction is mediated by a conserved Mlh1-interacting motif (MIP box). Despite this, we did not find a clear role for Rad5 in the canonical MMR mutation avoidance pathway. The interaction of Rad5 with Msh2 and Mlh1 is conserved in humans, although each of the Rad5 human homologs, HLTF and SHPRH, shared only one of the interactions: HLTF interacts with MSH2, and SHPRH interacts with MLH1. Moreover, depletion of SHPRH, but not HLTF, results in a mild increase in resistance to alkylating agents although not as strong as loss of MMR, suggesting gene duplication led to specialization of the MMR-protein associated roles of the human Rad5 homologs. These results provide insights into how MMR accessory factors involved in the MMR-dependent apoptotic response interact with the core MMR machinery and have important health implications into how human cells respond to environmental toxins, tumor development, and treatment choices of tumors with defects in Rad5 homologs.

## 1 Introduction

DNA mismatch repair (MMR) is the post-replicative repair pathway that repairs base-base mispairs and small insertion/deletion mispairs arising from DNA replication errors (Li, 2008; Fishel, 2015). MMR also induces apoptosis after recognizing mispairs induced by exogenous DNA damaging agents, such as O^6^-methylguanine:thymidine mispairs that occur after exposure to S_N_1 alkylators (Fu et al., 2012; Li et al., 2016). These lesions cannot be normally repaired by MMR as the O^6^-methylguanine lesion is on the template strand. Defects in the MMR result in an accumulation of mutations, which can result in altered cellular function and the development of cancers (Kolodner, 1995). Germline mutations in MMR genes are the underlying cause of the familial cancer predisposition syndrome, Lynch syndrome (Fishel et al., 1993; Lynch et al., 2015) and constitutional mismatch repair deficiency (Durno et al., 2015). Lynch syndrome predisposes individuals to several cancer types, primarily colorectal, stomach, endometrial, and ovarian cancers (de la Chapelle, 2004; Kastrinos and Stoffel, 2014), and constitutional mismatch repair deficiency is associated with many cancer types in pediatric patients (Durno et al., 2015). Somatic mutations and epigentic silencing in MMR genes are also found in a significant subset of sporadic cancers of the same subtypes (Borresen et al., 1995; Kane et al., 1997).

Mutation avoidance by eukaryotic MMR involves several steps: 1) mispair recognition by the heterodimeric MutS homologs, MSH2-MSH6 or MSH2-MSH3, 2) recruitment of the MutL homolog, MLH1-PMS2 (called Mlh1-Pms1 in *Saccharomyces cerevisiae*), 3) removal of the mispaired DNA from the daughter strand through either Exonuclease 1 (Exo1)-dependent, Rad27-dependent, or Exo1- and Rad27-independent MMR, and 4) gap-filling by the replicative polymerases, PCNA, and RFC, and 5) nick ligation (Li, 2008; Goellner et al., 2015; Fishel, 2015; Calil et al., 2021).

While the core machinery of eukaryotic DNA MMR is well defined, new MMR-interacting proteins are still being identified (Yuan et al., 2004; Li et al., 2013; Traver et al., 2015; Goellner et al., 2018; Terui et al., 2018; Rikitake et al., 2020; Calil et al., 2021). Remarkably, short peptide sequences have been identified that mediate interactions with Mlh1 (the Mlh1-interacting peptide motif or MIP box (Dherin et al., 2009)) and more recently Msh2 (the Msh2-interacting peptide motif or SHIP box (Goellner et al., 2018)). Together these motifs are involved in the interaction of *S. cerevisiae* Mlh1 with Ntg2, Sgs1, and Exo1, *S. cerevisiae* Msh2 with Exo1, Fun30, and Dpb3, and likely human MSH2 with SMARCAD1 (*S. cerevisiae* Fun30), WDHD1, and MCM9 (Dherin et al., 2009; Gueneau et al., 2013; Traver et al., 2015; Chen et al., 2016; Goellner et al., 2018). Identifying novel MMR accessory proteins and elucidating the mechanisms by which they interact with MMR will be critical to understanding mechanisms suppressing cancer development and potentially guiding cancer therapies involving DNA damaging agents.

Here we identify another novel MMR interacting partner, Rad5, that we predict to have both SHIP box and MIP box motifs. Rad5 is a helicase and E3 ubiquitin ligase involved in post-replication repair (PRR) pathways, which allow tolerance of template strand lesions that would otherwise lead to replication fork stalling (Xu et al., 2016; Gallo et al., 2019); however, Rad5 has no known role in MMR. PRR bypasses DNA template lesions via the error-prone translesion synthesis (TLS) and error-free template switching (TS) pathways (Gallo and Brown, 2019), the choice of which is in part controlled by the ubiquitination status of proliferating cell nuclear antigen (PCNA) (Gallo et al., 2019). The Rad5 E3 ligase has been associated with TS through the activity of Mms2-Ubc13-Rad5 in forming a lysine 63-linked polyubiquitination chain on PCNA (Motegi et al., 2008). However, recent studies have also identified Rad5 as a player in TLS through its interaction with the TLS protein Rev1 (Xu et al., 2016), which is consistent with the lack of epistasis of *rad5Δ* and *ubc13Δ* mutations observed in assays for genome instability (Putnam et al., 2010).

Rad5 has two known human homologs, Helicase-Like Transcription Factor (HLTF) and SNF2 Histone Linker PHD Ring Helicase (SHPRH). Both HLTF and SHPRH share the SNF2 helicase and RING finger domains with Rad5, and HLTF additionally shares the HIRAN (HIP116, Rad5 N-terminal) domain that is present N-terminal to the SNF2 helicase domain (Unk et al., 2010). Both HLTF and SHPRH have E3 ubiquitin ligase ability, both can polyubiquitinate PCNA, and HLTF can complement UV sensitivity of a *rad5Δ S. cerevisiae* strain (Unk et al., 2006; Unk et al., 2008; Masuda et al., 2012). HLTF and SHPRH also have direct but distinct roles in directing TLS- and TS-mediated PRR, and HLTF and SHPRH deletion mutants have different sensitivities to agents that cause DNA lesions (Seelinger et al., 2020). HLTF enhances TLS and inhibits SHPRH following UV damage, but MMS treatment instead causes SHPRH response and HLTF degradation (Lin et al., 2011). Loss of HLTF expression has been associated with several cancer types, including colorectal cancer (Moinova et al., 2002). Loss of SHPRH has also been associated with multiple cancers *via* 1) loss of heterozygosity of the long arm of chromosome 6, where SHPRH resides, 2) accumulation of SHPRH point mutations in melanoma and ovarian cancer-derived cell lines (Sood et al., 2003), and 3) through the protective action of a circular RNA encoding a 146 amino acid fragment of SHPRH in glioblastoma (Begum et al., 2018; Zhang et al., 2018).

In this study we confirm the predicted interactions in *S. cerevisiae* between Msh2 and Rad5 and between Mlh1 and Rad5 and verify that the Mlh1-Rad5 interaction is mediated by a MIP box. These interactions are conserved with human homologs HLTF and SHPRH. Interestingly, the Msh2-Rad5 and Mlh1-Rad5 interactions seem to have become split between the two homologs, with HLTF only binding to human MSH2 and SHPRH only binding to human MLH1. We also show that loss of SHPRH results in moderate resistance to alkylating agents. Together these data identify novel interacting partners of MMR in both yeast and humans and suggest that the SHPRH-MLH1 interaction is partially involved in an apoptotic response to damage-induced mispairs.

## 2 Materials and Methods

### 2.1 Chemicals and Reagents

Antibodies used in this study include anti-MLH1 (Cell Signaling Technologies 3515S), MSH2 (Cell Signaling Technologies 2017S), HLTF (Fisher PA5-30173), SHPRH (Santa Cruz sc-514395), IgG (Santa Cruz sc-2025). 6-Thioguanine (6 TG) was obtained from TCI America (T0212-1G) delivered by VWR, and MNNG was obtained from Sigma-Aldrich (Cat #129941).

### 2.2 Two-Hybrid Assay

Plasmids expressing fusion proteins for yeast two-hybrid analysis were generated by Gateway cloning (Invitrogen) the gene of interest without its start codon into either the Gateway-modified bait vector, pBTM116, which encodes the LexA DNA binding domain and Trp1, or the Gateway-modified prey vector, pACT2, which encodes the GAL4 activation domain and Leu2. Bait and prey plasmids were co-transformed into the L40 *S. cerevisiae* reporter strain L40 (*MAT* **a** *trp1-901 leu2-3112 his3Δ200 LYS2(4lexAop-HIS3) URA3(8lexAop-lacZ)*), in which a positive interaction of the bait and prey fusion proteins results in expression of *HIS3* and hence complementation of the *his3Δ200* mutation (Yan and Jin, 2012). Colonies were grown overnight in complete synthetic medium lacking leucine and tryptophan (CSM -Leu -Trp) to maintain plasmid selection and then 10-fold serial dilutions were spotted onto CSM -Leu -Trp control medium and CSM -Leu -Trp -His selective medium to assay for two-hybrid interactions.

### 2.3 Mutation Rate and Mutation Spectra Analysis

*S. cerevisiae* strains were grown in YPD (1% yeast extract, 2% Bacto Peptone and 2% dextrose) or in the appropriate synthetic dropout media (0.67% yeast nitrogen base without amino acids, 2% dextrose, and amino acid dropout mix at the concentration recommended by the manufacturer (US Biological) at 30 °C. All *S. cerevisiae* strains in this study were derived in the S288c strain background using standard gene deletion and pop-in, pop-out methods.

Mutator phenotypes were evaluated using the *hom3-10* frameshift reversion assay. Mutation rates were determined by fluctuation analysis using a minimum of 2 independently derived strains and 14 or more independent cultures; comparisons of mutation rates were evaluated using 95% confidence intervals.

One independent Thr^+^ revertant was isolated per culture from fluctuation tests. Chromosomal DNA was isolated from each revertant using a Qiagen Puregene Yeast/Bact. Kit B and the *hom3-10* region were amplified by PCR using the Primer 5′-AGT​TGT​TTG​TTG​ATG​ACT​GC and Primer 5′-TTC​AGA​AGC​TTC​TTC​TGG​AG and sequenced with the Primer 5′-CTT​TCC​TGG​TTC​AAG​CAT​TG using a commercial sequencing facility (Calil et al., 2021).

### 2.4 Bioinformatic Analyses

Bioinformatic analysis of potential MIP and SHIP motifs with good peptide matching scores in regions predicted to be unstructured was carried out as described previously (Goellner et al., 2018). Briefly, we determined the count of each amino acid at each position in the alignment of the SHIP boxes 1 and 2 or the MIP box from fungal Exo1 homologs. A pseudocount of 1 was added to all positions that were zero, and then the counts were converted to a fraction, *F*_*k,j*_, for each amino acid *k* at position *j*. *F*_*k,j*_, values were then converted to log probabilities (*M*_*k,j*_) scaled by a background model: *M*_*k,j*_ = log*(F*_*k,j*_ */b*_*k*_ *)*. The background model was calculated using the frequency of the different amino acids in the proteins encoded by the *S. cerevisiae* genome. Raw scores (*S*_*raw*_) for peptides were calculated by adding up all *M*_*k,j*_ values from the PSSM for each amino acid *k* at position *j* within the peptide sequence. We scaled the raw scores to be in the range 0–1 using the equation: *S*_*scale*_ = *(S*_*raw*_ − *S*_min_ *)/(S*_max_ − *S*_min_ *)*, where *S*_min_ and *S*_max_ are the minimum and maximum scores possible for any peptide scored by the PSSM. The long-term disorder prediction score for each position in the proteins were generated using IUPRED (Dosztanyi et al., 2005), and the disorder prediction score for each peptide was calculated by averaging the scores for each of the residues in the peptide.

Identification of Rad5 homologs for the analysis of the conservation of the candidate MIP and SHIP motifs was performed by categorizing BLAST hits from each species by building a phylogenetic tree with MAFFT version 7.305 (Katoh and Standley, 2014) and PHYLIP version 3.696 (Retief, 2000) that contained all of the BLAST hits from that species with all of the *S. cerevisiae* Rad5 homologs (Chd1, Fun30, Ino80, Irc20, Irc5, Isw1, Isw2, Mot1, Rad16, Rad26, Rad5, Rad54, Rdh54, Snf2, *S*th1, Swr1, and Uls1). Homologs were then assigned if the BLAST hit was on the same branch as the phylogram as only one of the *S. cerevisiae* reference sequences using the program idwtree (Goellner et al., 2018). Alignments of assigned fungal Rad5 homologs were then performed with MAFFT for analyzing conservation and building sequence logos with Seq2Logo (Thomsen and Nielsen, 2012).

### 2.5 Cell Culture

All cell lines were cultured at 5% CO_2_ and 37°C. Hek293 and Hek293T cells were cultured in DMEM supplemented with 10% FBS (Gibco Life Technologies Corporation) and 1% Penicillin/Streptomycin (Gibco, Life Technologies). HeLa S3 cells were cultured in RPMI supplemented with 10% FBS and 1% Penicillin/Streptomycin.

### 2.6 Generation of Knockout Lines

HeLa MLH1, MSH2, HLTF, and SHPRH knockout cell lines and the HLTF and SHPRH double knockout cell line were generated by CRISPR-Cas9 technologies, using single guide RNA (sgRNA) sequences (Table 1) for each of the genes listed. The LentiCRISPRv2 was a gift from Feng Zhang (Addgene plasmid #52961). The plasmid was digested with BsmBI and gel purified using the QIAquick PCR purification kit according to the manufacturer’s instructions. Complementary oligonucleotides (synthesized by Integrated DNA Technologies) encoding the sgRNA were then annealed and cloned into LentiCRISPRv2. Cells were then transfected with Lipofectamine 3000 (Thermo Scientific L3000008) and the cells were selected with puromycin (Promega). Single cell clones were allowed to grow up under puromycin selection and expanded. Loss of protein expression was confirmed for each clone using SDS-PAGE and western blot analysis.

**TABLE 1:** sgRNA sequences for knockout cell line generation.

Name	Forward Primer	Reverse primer
sgHLTF+2	5′-CAC​CGG​TTG​GAC​TAC​GCT​ATT​ACA​C-3′	5′-AAA​CGT​GTA​ATA​GCG​TAG​TCC​AAC​C-3′
sgSHPRH +1	5′-CAC​CGC​TGG​AGG​AGC​ACG​TTT​CCG​T-3′	5′-AAA​CAC​GGA​AAC​GTG​CTC​CTC​CAG​C-3′
sgSHPRH -2	5′-CAC​CGT​TGT​GAC​AAG​GGT​ATT​CTG​G-3′	5′-AAA​CCC​AGA​ATA​CCC​TTG​TCA​CAA​C-3′
sgMLH1 -1	5′-CAC​CGT​GAT​AGC​ATT​AGC​TGG​CCG​C-3′	5′-AAA​CGC​GGC​CAG​CTA​ATG​CTA​TCA​C-3′
sgMSH2 +4	5′-CAC​CGC​TTC​TAT​ACG​GCG​CAC​GGC​G-3′	5′-AAA​CCG​CCG​TGC​GCC​GTA​TAG​AAG​C-3′

### 2.7 Short-Term Cytotoxicity Assay

HEK293 cells were plated at 750,000 cells/well in a 6-well plate 24 h prior to transfection. Cells were transfected with siHLTF (Origene) or siSHPRH (Origene) alone or in combination utilizing the Lipofectamine RNAiMAX Transfection Reagent (Invitrogen). After transfection for 24 h, the cells were seeded at 10,000 cells/well in 96-well plates, and the remaining cells were collected for protein analysis. Media was removed 24 h after seeding and cells were treated with the indicated doses of MNNG for 1 h. The media was then replaced and allowed to grow for 72 h, at which time cell viability was measured using the CellTiter 96 Aqueous One Solution Cell Proliferation Assay (MTS) kit (Promega) according to the manufacturer’s instructions.

### 2.8 Long-Term Clonogenic Cytotoxicity Assay

HEK293 or HeLa S3 cells were plated in a 6-well plate 24 h prior to treatment. Cells were pre-treated with 10 μM O^6^-benzylguanine (6-(benzyloxy-7H-purin-2-amine, Thermo Scientific, H60274-MD) for 2 h and then pulsed with MNNG or DMSO vehicle control for 1 h. Cells were then trypsinized and plated in a 6-well plate at a density of 300 cells/well for HeLa or 3,000 cells/well for HEK293 with normal media and were allowed to grow for 10 days, or until colonies of approximately 50 cells could be seen. The cells were then stained with crystal violet and the number of colonies were counted.

### 2.9 Nuclear Protein Extraction

Cells were washed with PBS, resuspended in cytoplasm extract buffer (20 mM Hepes, 10 mM KCl, 0.1 mM EDTA, 1 mM DTT, and protease inhibitor) and then chilled on ice for 10 min. 0.75% Nonidet P-40 (NP-40) lysis buffer was added and the solution was pipetted to mix followed by vortex mixing for 10 s. The cells were centrifuged at 800 x g for 3 min at 4°C to separate nuclei from cytoplasm (supernatant). The cytoplasm extract was placed in a separate tube and the nuclei pellet was resuspended in 25% sucrose/cytoplasm extraction buffer and pipetted to disperse. The cells in 25% sucrose/cytoplasm extraction buffer were underlaid with half the volume of 50% sucrose/cytoplasm extraction buffer and centrifuged at 10,000 x g for 15 min at 4°C. The supernatant was removed, and the nuclei pellet was lysed in PBE150Na (50 mM Tris-HCl at pH 7.5, 1 mM ethylenediaminetetraacetic acid (EDTA) at pH 8.0, 150 mM NaCl, 0.5% sodium deoxycholate and 1% NP-40, containing 1x complete protease inhibitor cocktail (Roche Diagnostics GmbH, Germany)). The pellet was then sonicated and centrifuged at 10,000 x g for 15 min at 4°C. The supernatant was collected as the nuclear extract.

### 2.10 Immunoprecipitation

Co-immunoprecipitations of endogenous or tagged proteins were performed using magnetic protein A/protein G beads (Thermo Scientific) followed by a conjugation step to either the IgG control or antibody of interest with BSA for 2 h followed by washes. Conjugated beads were incubated with whole cell lysate or nuclear extracts (described above) at 4°C overnight rotating followed by increasing salt washes. Beads were boiled with 6x loading buffer and samples were run on SDS-PAGE gels followed by western blot.

### 2.11 HPRT Mutagenesis Assay

The HPRT forward mutagenesis assay was performed in HeLa S3 and HeLa S3 knockout cells as described previously (Li et al., 2013). Cell lines were first cultured in hypoxanthine, aminopterin, and thymidine (HAT) supplemented media (ThermoFisher Scientific, supplied as 50x supplement) for at least five passages to clear background HPRT mutations. HAT passaged cells were seeded at 5 × 10^5^ cells per 100 mm dish in triplicate, allowed to adhere overnight, then treated with 5 µM 6-thioguanine (6-TG). Plating efficiency of the cells was determined by culturing 5 × 10^2^ HAT passaged cells per 100 mm dish plated in triplicate in the absence of 6-TG. The media was replaced every 2 to 3 days. After 10 days of culturing cell colonies were stained with 0.5% crystal violet in 25% methanol and the colonies containing more than 50 cells were counted. Mutation frequency was determined by calculating the median for mutant cells (number 6-TG selected colonies/5 × 10^5^ cells plated) and the median for plating efficiency (number untreated colonies/5 × 10^2^ cells plated) and dividing mutation by plating efficiency for each cell line.

### 2.12 Cell Synchronization

Cell synchronization was conducted by performing a double thymidine block in HeLa cells. The protocol for double thymidine block was adapted from a previous publication (Schroering and Williams, 2008). HeLa cells were plated and after 1 day, washed once with warmed PBS and cultured in complete medium containing 2 µM thymidine (Sigma T9250) for 18 h. The HeLa cells were washed twice with warmed PBS and released in media without thymidine for 9 h. The cells were then cultured with 2 µM thymidine for 16 h, washed once with warmed PBS and replaced with fresh media for collection at each of the times indicated for each figure. For treated cells, cells were released into complete media containing 0.2 µM MNNG or DMSO containing O^6^-benzylguanine.

### 2.13 Cell Cycle Analysis

After cell synchronization using the double thymidine block, cells were trypsinized and quenched with media then centrifuged at 2,000 rpm for 5 min. The cells were then resuspended in 75% ethanol for at least 1 h at −20°C for fixation. The cells were centrifuged at 4,000 rpm for 2 min and then resuspended in PBS containing 0.25% Triton X-100 for 15 min. Cells were centrifuged at 4,000 rpm for 2 min and resuspended in PBS containing 10 μg/ml RNase A (Qiagen) and Propidium Iodide Ready Flow Reagent (ThermoFisher Scientific). Subsequent detection of the cell cycle phase distribution was accomplished by using propidium iodide for nuclear staining and detection using the BD FACSymphony A3 flow cytometer and collecting FSC, SSC, PE for propidium iodide, and BB515 for compensation with gating for single cells. The resulting data was analyzed by FlowJo software.

### 2.14 Statistical Analysis

Calculations of the mean, standard error, statistical analysis, and comparison of each set of experimental means was performed with Graphpad Prism 9.0 (Graphpad Software Inc., La Jolla, CA, United States ).

## 3 Results

### 3.1 Rad5 Physically Interacts With Yeast Mlh1 and Msh2

**FIGURE 1:**
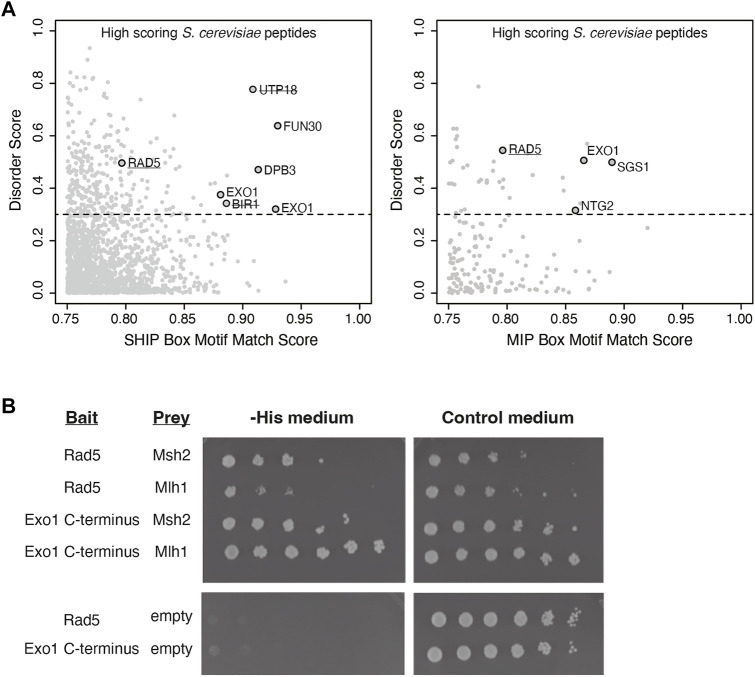
Rad5 has a predicted MIP and SHIP box and interacts with Mlh1 and Msh2. **(A)** The match score of 1,745 peptides from the nuclear *S. cerevisiae* proteome with a moderate or good motif matching to either the MIP or SHIP box motif as determined from bioinformatic analysis using a position-specific scoring matrix (PSSM) are plotted against their long-range disorder predicted by IUPRED (Dosztanyi et al., 2005). Rad5 was identified in analysis for both MIP and SHIP motifs. **(B)** Yeast two-hybrid analysis shows both Msh2 and Mlh1 prey constructs interact with Rad5 bait (growth on–Leu–Trp–His selective medium as well growth on the control–Leu–Trp medium). Exo1-C terminus bait shows positive interactions with Msh2 and Mlh1 prey as a positive control. Neither Rad5 or Exo1-C terminus bait constructs autoactivate in the presence of an empty prey vector.

To identify candidate MMR-interacting proteins, we computationally screened the *S. cerevisiae* proteome for proteins containing sequences resembling MIP and SHIP box motifs following our previous strategy that identified the Msh2-interacting and SHIP box-containing proteins Fun30 and Dpb3 (Goellner et al., 2018). First, the MIP motif match score and the SHIP motif match score were calculated for every 7 amino acid peptide computationally generated from the *S. cerevisiae* S288c proteome using a position-specific scoring matrix (PSSM (Stormo et al., 1982)) derived from an alignment of 301 fungal Exo1 MIP box sequences and a PSSM from an alignment of 566 fungal Exo1 SHIP box sequences. Second, high-scoring hits were filtered for proteins known to be in the nucleus or with an unknown cellular localization. Third, the average disorder score for each peptide was determined by the averaging the long-range disorder score for the 7 amino acids of the peptides after analysis of the relevant proteins for long-range disorder with IUPRED (Dosztanyi et al., 2005). The motif match scores were then plotted against the average disorder scores (Figure 1A) to identify candidate peptides that matched the MIP box consensus or the SHIP box consensus and that were in disordered protein regions. These analyses identified proteins containing known functional MIP boxes (Exo1, Ntg2, Sgs1) and known functional SHIP boxes (Exo1, Fun30, Dpb3) in unstructured protein regions as well as high scoring SHIP box-like peptides in proteins previously demonstrated as not interacting with Msh2 (Utp18, Bir1) (Figure 1A (Goellner et al., 2018)). These analyses also implicated a candidate MIP box sequence and a candidate SHIP box sequence in the Rad5 protein.

Because this analysis suggested that Rad5 resembled Exo1, which also has both MIP and SHIP box motifs and uses both of these motifs for recruitment to MMR (Goellner et al., 2018), we sought to confirm the predicted Rad5 interactions using yeast two-hybrid analysis. We generated a bait plasmid containing *S. cerevisiae* Rad5 fused to the LexA DNA-binding domain. This plasmid, a positive control bait plasmid encoding the Exo1 C-terminus fused to LexA, or a negative control empty bait plasmid encoding only the LexA DNA-binding domain were then cotransformed into the *S. cerevisiae* tester strain L40 with prey plasmids that encoded *S. cerevisiae* Mlh1 or Msh2 fused to the Gal4 transcriptional activation domain. In the L40 tester strain, physical interaction between the bait and prey proteins drives expression of the *HIS3* gene and hence supports growth on medium lacking histidine. As expected, the yeast two-hybrid analysis revealed an interaction between the Exo1 C-terminus and both the Mlh1 and Msh2 prey vectors. The Rad5 bait plasmid also supported growth on -His medium in combination with both the Mlh1 and Msh2 prey vectors, but not the empty prey vector (Figure 1B), indicating that Rad5 can interact with both Mlh1 and Msh2.

### 3.2 Rad5 Binds to Mlh1 Through the MIP Box Motif

**FIGURE 2:**
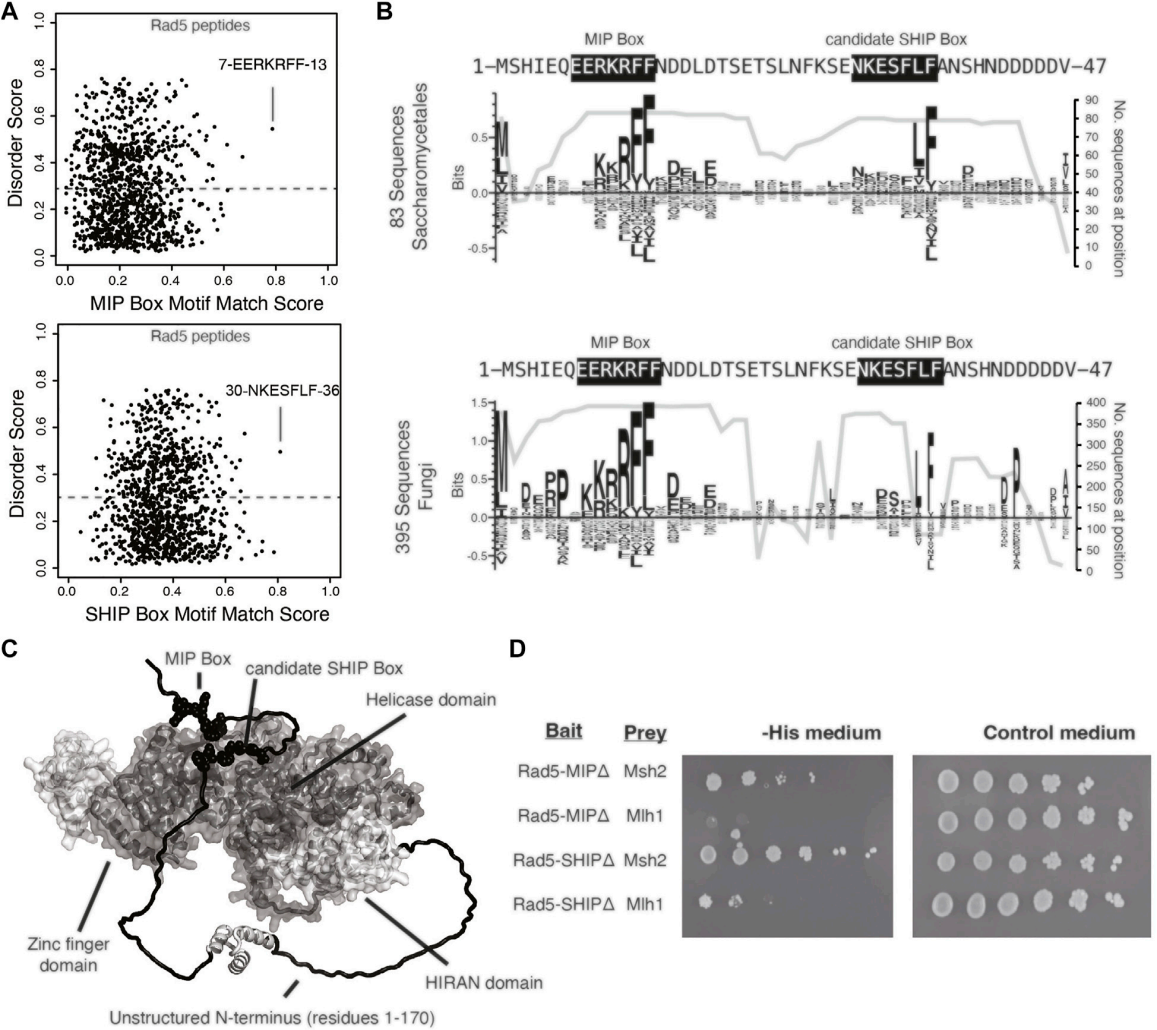
Rad5 interacts with Mlh1 through a MIP box motif but does not interact with Msh2 through a SHIP box motif. **(A)** The MIP box and SHIP box motif matching scores for every 7mer peptide in *S. cerevisiae* Rad5 is plotted against the predicted disorder score, showing that the predicted MIP and SHIP boxes have the best peptide scores and are predicted to be disordered by IUPRED (Dosztanyi et al., 2005). **(B)** Sequence logos of the first 47 amino acids of *S. cerevisiae* Rad5 generated by Seq2Logo (Thomsen and Nielsen, 2012) were calculated from an alignment of 83 Saccharomycetales Rad5 sequences (top) or 395 fungal Rad5 sequences (bottom). Large letters above the zero line correspond to highly conserved residues in the alignment. The number of sequences with residues at this location is plotted underneath the sequence logo; note that the MIP box is present in almost all fungal Rad5 sequences aligned whereas the candidate SHIP box is present only in a small subset of the fungal Rad5 sequences corresponding to the Saccharomycetales (bottom). **(C)** Mapping of the predicted MIP and SHIP motifs (black spheres) onto the Alphafold2-predicted structure of *S. cerevisiae* Rad5 (Jumper et al., 2021; Varadi et al., 2022) reveals that these predicted motifs are in the unstructured N-terminus (black). **(D)** Yeast two-hybrid analysis shows that mutation of the predicted MIP box in the Rad5 bait vector retained the interaction with the Msh2 prey but resulted in a loss of interaction (indicated by no growth on selective -Leu -Trip -His medium) with the Mlh1 prey vector, whereas mutation of the predicted SHIP box in the Rad5 bait vector retained interaction with both the Msh2 and Mlh1 prey vectors.

To gain insight into the Rad5 interactions with Mlh1 and Msh2, we sought to determine if these interactions were mediated through the predicted MIP box (peptide 7-EERKRFF-13) and the predicted SHIP box (peptide 30-NKESFLF-36), which are in the unstructured N-terminus of Rad5 (Figures 2A,C). Analysis of the conservation of these predicted motifs revealed that the predicted MIP box is extensively conserved in all fungi, whereas the predicted SHIP box is restricted to fungi in the order Saccharomycetales, which includes *S. cerevisiae* (Figure 2B). We and others have previously shown that mutating the conserved phenylalanine and tyrosine amino acids in these motifs to alanine disrupts the ability of these motifs to mediate interactions (Dherin et al., 2009; Goellner et al., 2018). We therefore mutated the predicted Rad5 MIP motif 7-EERKRFF-13 to 7-EERKRAA-13 (Rad5-MIPΔ) and the predicted SHIP motif 30-NKESFLF-36 to 30-NKESALA-36 (Rad5-SHIPΔ) in our Rad5 yeast two-hybrid bait plasmid. Yeast two-hybrid analysis demonstrated that the Rad5-MIPΔ mutant binds to Msh2 but not to Mlh1, indicating the Rad5-Mlh1 interaction, but not the Rad5-Msh2 interaction, is mediated by the predicted MIP box motif (Figure 2D). In contrast, the Rad5-SHIPΔ mutant bound to both Mlh1 and Msh2 (Figure 2D), indicating that the Rad5-Msh2 interaction involves another region of Rad5, an extended SHIP box that requires additional mutations to disrupt, or redundant interactions with either the putative SHIP box or another region of Rad5.

### 3.3 Loss of *RAD5* Causes a Minor Increase in Mutation Rate and a Mutation Spectrum That Is Not Representative of That Caused by a MMR Defect

**FIGURE 3:**
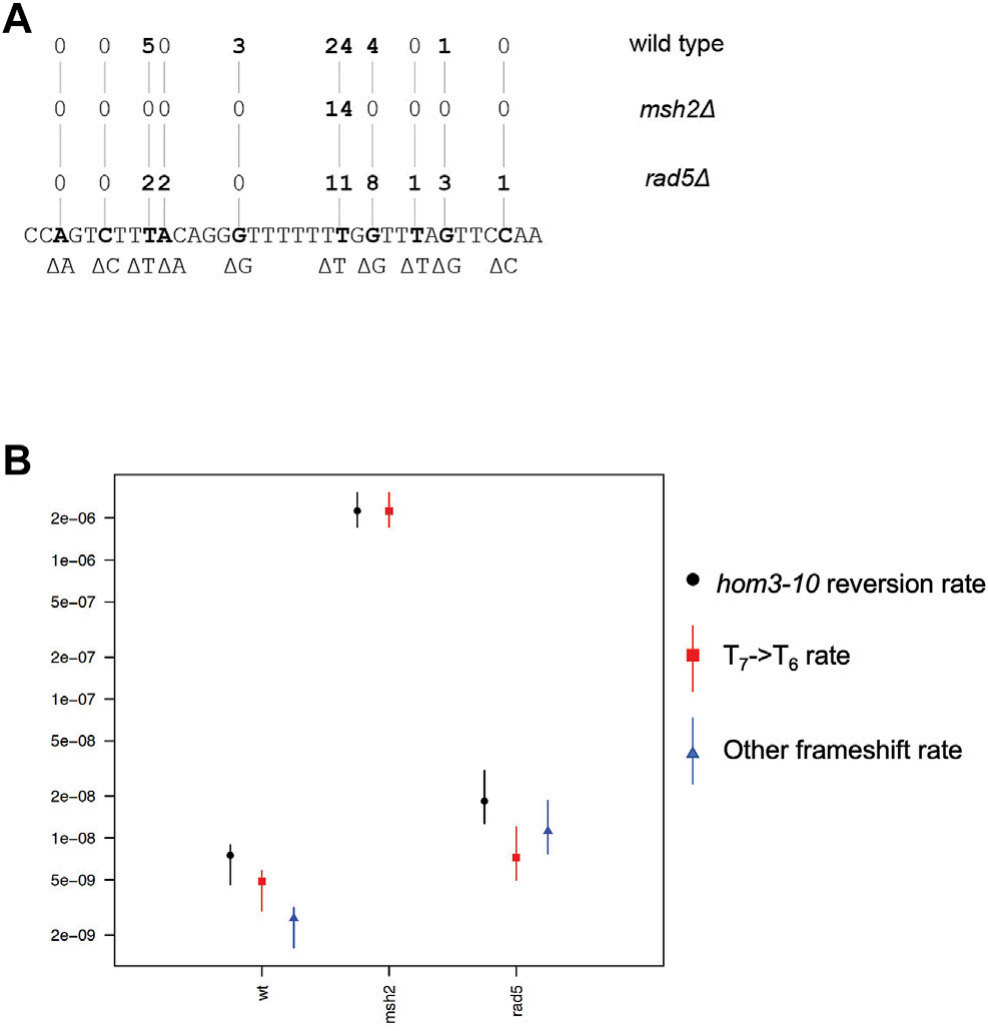
Rad5 deletion strain has an altered mutation spectra from MMR deficient strains. **(A)** Spectrum of mutations selected in the *hom3-10* frameshift reversion assay that measures 1 base pair frameshifts in the modified *HOM3* gene that is required for the synthesis of threonine. 37 isolates were analyzed for the WT strain, 14 isolates were analyzed for the *msh2Δ* strain, and 28 isolates were analyzed for the *rad5Δ* strain. MMR deficient strains primarily have T7→ T6 frameshifts. MMR proficient strains have more non T7 → T6 reversion isolates. *rad5Δ* mutation spectrum resembles a WT strain more than a MMR deficient strain. **(B)** Graph of portion of overall *hom3-10* mutation rate made up of T7 → T6 reversions or non T7 → T6 reversions. Overall mutation rate for each strain is in black. Proportion of the rate represented by T7 → T6 reversion rate is in red.

Given that Rad5 binds to Msh2 and Mlh1, we investigated if loss of *RAD5* gave rise to a MMR defect in the absence of DNA damage by determining the mutation rate of a *RAD5* deletion strain with the *hom3-10* frameshift reversion assay. In the *hom3-10* assay, -1 frameshift mutations restore growth on medium lacking threonine. An *MSH2* deletion strain, which is completely deficient for MMR, had a 336-fold increase in mutation rate over the wild-type strain. However, the *rad5Δ* strain only had a 2.5-fold increase in mutation rate (Table 2). To determine whether this modest rate increase was representative of a defect in the canonical mutation avoidance MMR pathway, the *HOM3* gene was sequenced for 14–37 reversion isolates from each genotype (Figure 3A). MMR deficient strains result in almost entirely T7 → T6 frameshifts (Tishkoff et al., 1997; Flores-Rozas and Kolodner, 1998; Calil et al., 2021)), and consistent with this, 100% of the revertants from the *msh2Δ* strain were T7 → T6 frameshifts (Figure 3B). The wild type revertants had a wider variety of frameshift reversion mutations (only 65% T7 → T6 frameshifts), although at a much lower rate of occurrence (Figure 3B). The *RAD5* deletion strain had a mutation spectrum more similar to the wild-type strain with even more kinds of frameshifts observed (only 39% T7 → T6 frameshifts), which may reflect roles of *RAD5* in PRR and not MMR. Together these data suggest that loss of *RAD5* does not have a strong influence on canonical mutation avoidance pathway of MMR during unperturbed growth consistent with previous results (Johnson et al., 1992).

**TABLE 2:** *hom3-10* reversion rates.

Genotype	Strain	hom*3-10 reversion rate*
Wild type	RDKY6677	7.50 [4.61–8.95] x 10^−9^ (1)
*msh2Δ*	RDKY6696	2.52 [1.72–3.04] x 10^−6^ (336)
*rad5Δ*	RDKY6898	1.84 [1.27–3.06] x10^−8^ (2.5)

Reported rates are the median rates with 95% confidence interval in square brackets. Fold increase in mutation rate is listed in parenthesis as compared to the wild-type strain. n = 14–57 independent cultures from two independently derived isolates.

### 3.4 Human Homologs of Rad5, HLTF and SHPRH, Have Split Binding Between MSH2 and MLH1

**FIGURE 4:**
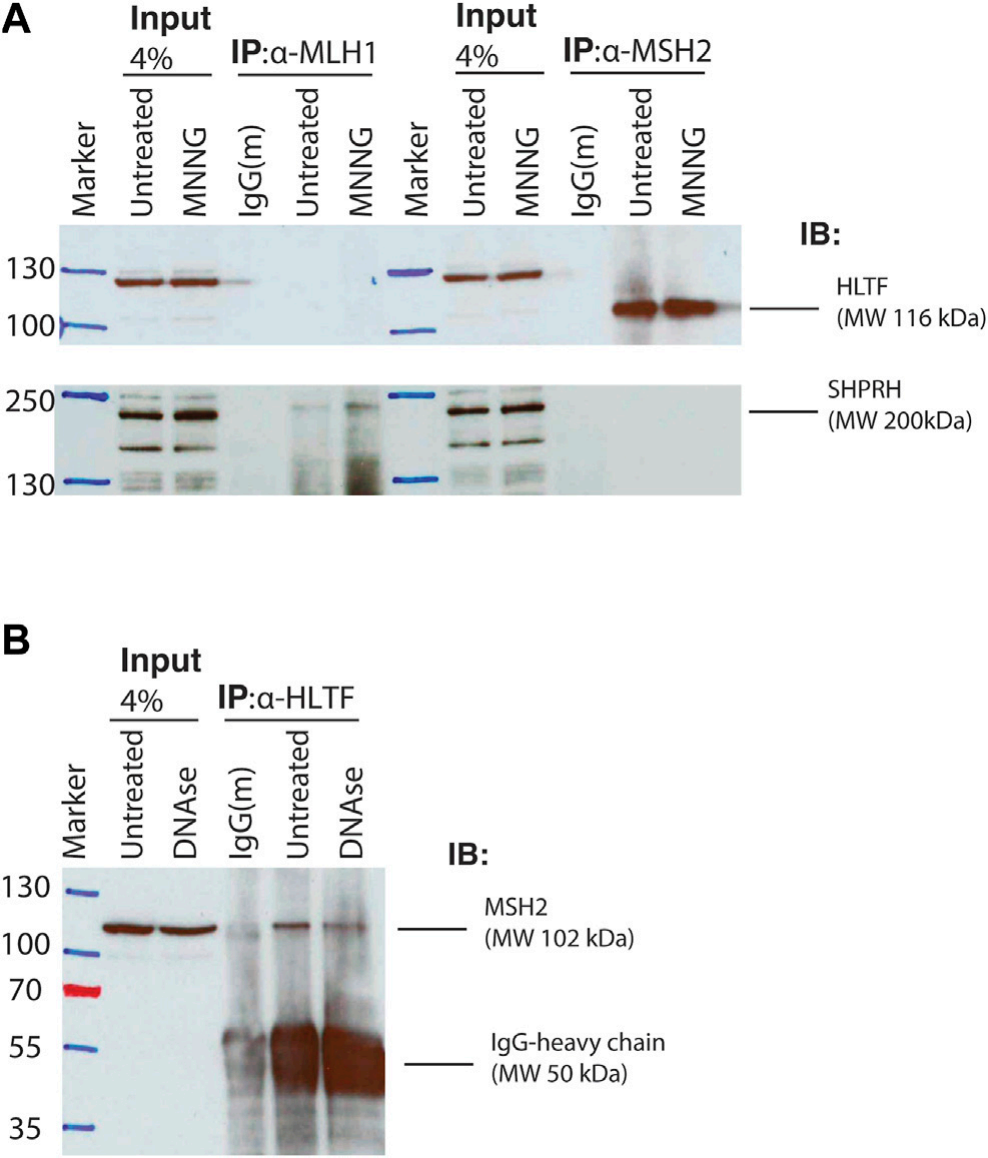
Human homologs of Rad5 HLTF and SHPRH interact with MSH2 and MLH1. **(A)** HeLa cells were treated with DMSO or 30 μM MNNG and lysates were fractionated to obtain the nuclear fraction. Nuclear fractions were immunoprecipitated with either anti-MLH1 or anti-MSH2 beads and immunoblotted for either HLTF and SHPRH. HLTF co-immunoprecipitated with MSH2. Immunoprecipitated HLTF runs at the predicted molecular weight of 116 kDa, however the non-immunoprecipitated HLTF in the input lanes runs at a slightly higher MW and has several additional bands consistent with the product sheet for the ThermoFisher HLTF antibody. SHPRH co-immunoprecipitated with MLH1 and the interaction is increased after MNNG treatment. **(B)** HeLa cell nuclear lysates were treated with or without DNAse. Nuclear fractions were obtained and immunoprecipitated with anti-HLTF beads and immunoblotted for MSH2. MSH2 interacts with HLTF regardless of DNAse treatment.

To test whether the interactions identified between Rad5 and the MMR proteins are conserved in humans, we used co-immunoprecipitation of nuclear fraction lysates from HeLa cells to detect interactions between MMR proteins and the Rad5 human homologs, HLTF and SHPRH. HeLa cells have proficient MMR and undergo MMR-mediated apoptosis after alkylating agents (Li et al., 2013; Takeishi et al., 2020). MSH2 directly interacted with HLTF (Figure 4A). This interaction was stable even after DNase treatment, indicating that the co-immunoprecipation was not simply through simultaneous association with DNA (Figure 4B). MSH2 and HLTF interacted constitutively in basal conditions and the interaction did not change when the DNA alkylating agent MNNG was added (Figure 4A). No co-immunopreciptation of HLTF with MLH1 was observed under either basal or DNA damaging conditions (Figure 4A). In contrast, we found that SHPRH co-immunopreciptated with MLH1 under basal conditions and that the interaction was enhanced by the presence of MNNG-induced DNA damage (Figure 4A). Unlike HLTF, SHPRH did not co-immunopreciptate with MSH2 under either basal or DNA damaging conditions (Figure 4A). Together this shows that the binding between Rad5 homologs and MMR proteins is conserved throughout evolution to human cells, and interestingly, the interactions with the core MMR proteins seem to be split between the two human Rad5 homologs.

### 3.5 HLTF Interacts Differently With Msh2 Than Other SHIP Box Containing Proteins

**FIGURE 5:**
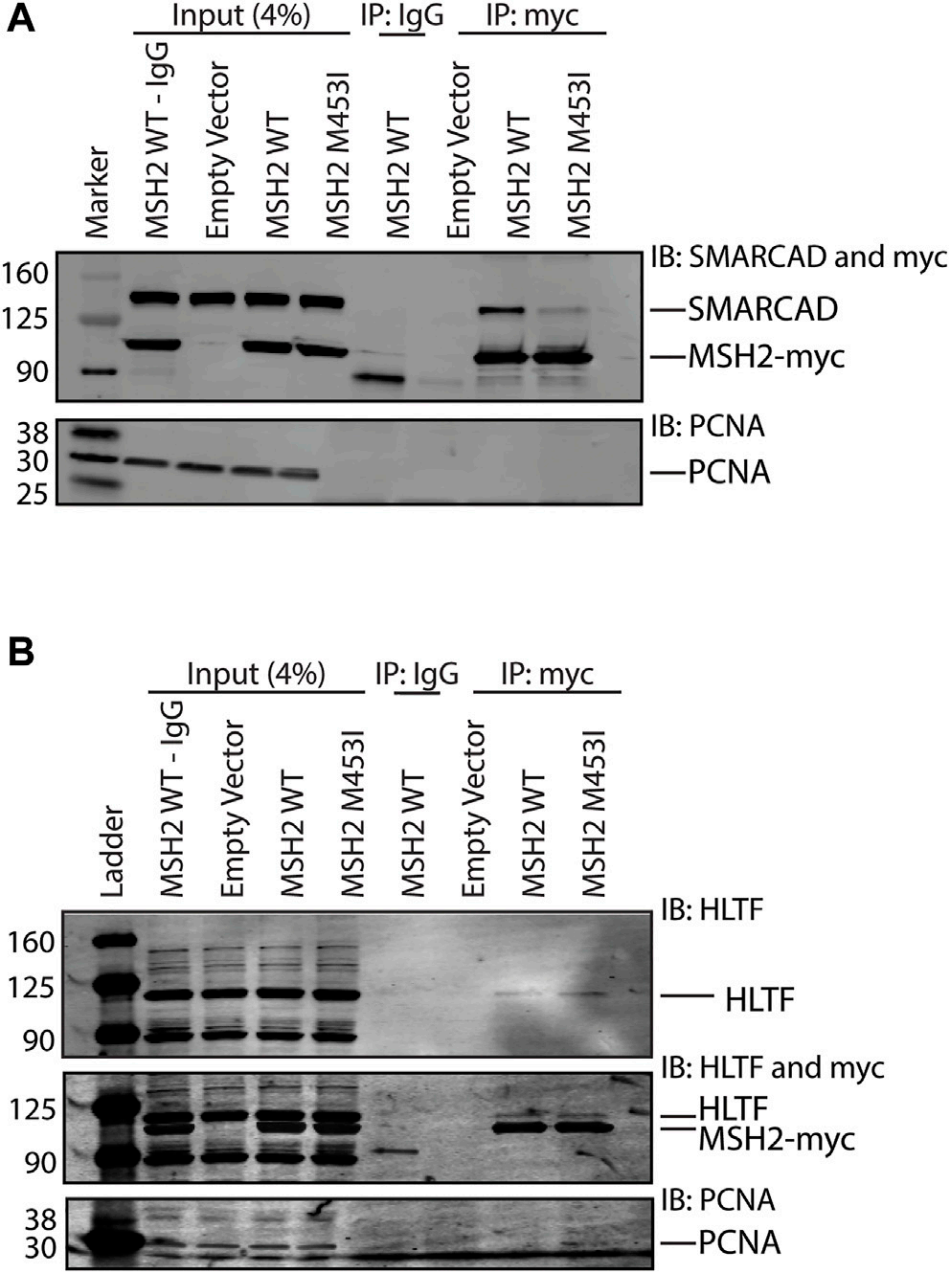
HLTF retains binding with the MSH2 M453I mutation. **(A)** HEK293T cells were transfected with c-terminal Myc-FLAG tagged MSH2 WT or MSH2-M453I mutant constructs. Myc-tagged MSH2 was immunoprecipitated with anti-Myc beads and immunoblotted for SMARCAD1. SMARCAD1 co-immunoprecipitated with MSH2 WT but not the MSH2 M453I hinge region mutation. **(B)** HEK293T cells were transfected with c-terminal Myc-FLAG tagged MSH2 WT or MSH2 M453I mutant constructs and Myc-tagged MSH2 was immunoprecipitated with anti-Myc beads and immunoblotted for HLTF. HLTF co-immunoprecipitated with MSH2 WT as we observed with endogenous protein co-IPs. HLTF also co-immunoprecipitated with Msh2 M453I unlike SMARCAD1.

Given that Rad5’s interaction with Msh2 could not be disrupted by mutation of the predicted SHIP box (Figure 2), we further investigated the human HLTF-MSH2 interaction. During the *S. cerevisiae* studies that identified the SHIP box motif, we also identified that the *msh2-M470I* mutation, which affects an amino acid in the hinge linker, disrupted the ability of Msh2 to bind to the SHIP box peptide (Goellner et al., 2018). To determine if HLTF interacted in a similar manner with MSH2, we generated the equivalent human mutation M453I in our myc-tagged MSH2 construct. We confirmed that the human mutation also disrupted SHIP box interactions by testing co-immunoprecipitation of MSH2 and MSH2-M453I with SMARCAD1 (Figure 5A). SMARCAD1 is the human homolog of *S. cerevisiae* Fun30; both SMARCAD1 and Fun30 contain a conserved N-terminal SHIP box. SMARCAD1 interacts with MSH2 in human and *Xenopus*, and the Fun30-Msh2 interaction in yeast is eliminated by the *msh2-M470I* mutation (Goellner et al., 2018; Terui et al., 2018; Takeishi et al., 2020). We found that SMARCAD1 interacts with wild-type MSH2 but has markedly reduced binding to the MSH2-M453I mutant (Figure 5A). In contrast, HLTF co-immunoprecipitated with both wild-type MSH2 and the MSH2-M453I mutant (Figure 5B). Taken together, evidence from both the *S. cerevisiae* Rad5-Msh2 interaction and the human HLTF-MSH2 interaction suggest that this interaction is distinct from those mediated by the SHIP box motif. Investigations into this mode of binding are ongoing.

### 3.6 SHPRH Interacts With MLH1 Only During S-phase

**FIGURE 6:**
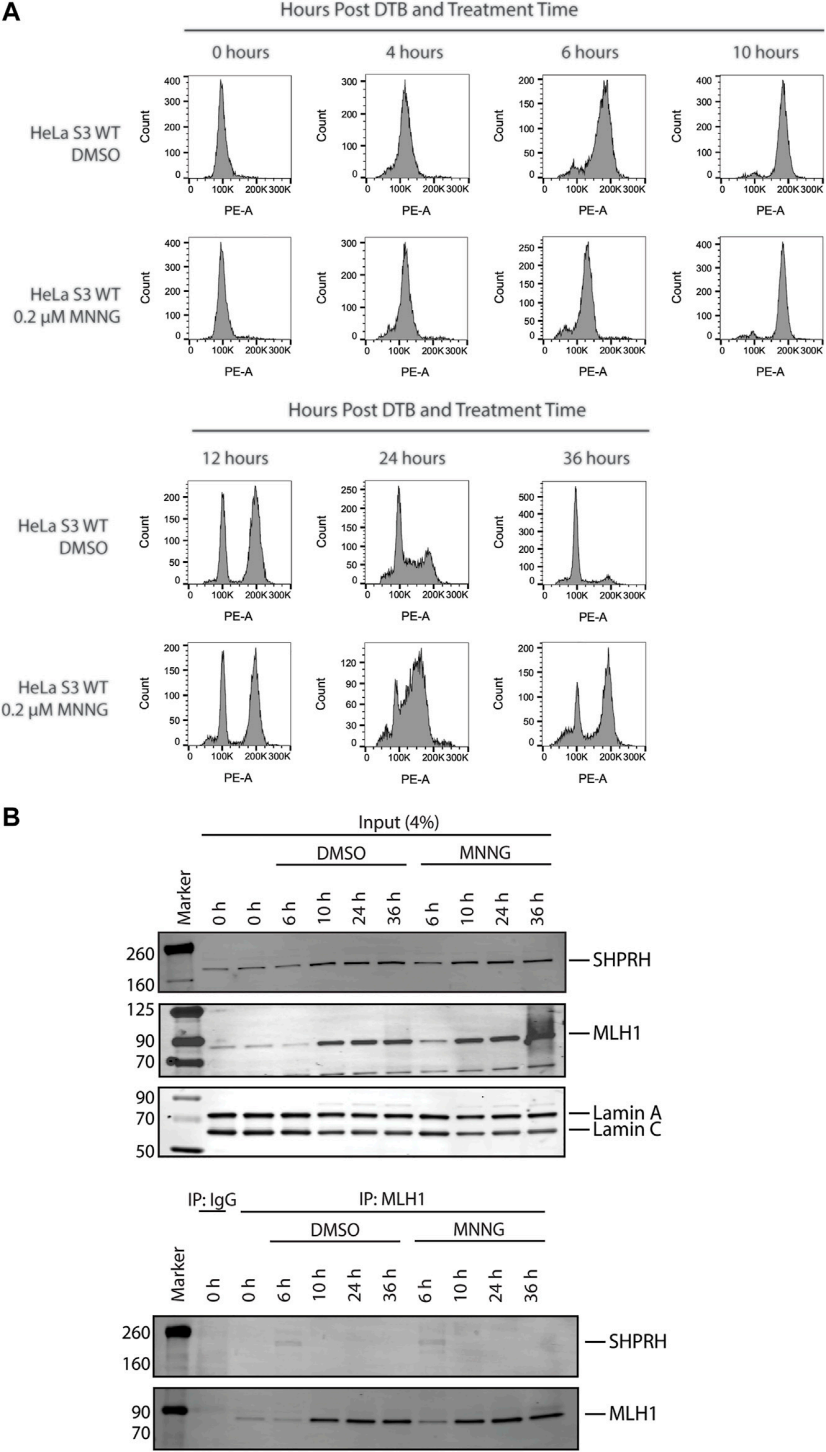
SHPRH interaction with MLH1 occurs within S phase of the cell cycle. **(A)** Cell cycle progression of HeLa WT cells treated with DMSO or 0.2 µM MNNG after release from double thymidine block (DTB) synchronization. HeLa WT cells have a G2/M arrest after the second cell cycle (24 h) following treatment with MNNG. The G2/M arrest does not occur in HeLa WT cells treated with DMSO. **(B)** HeLa WT cells were synchronized in the G0/G1 cell cycle utilizing DTB synchronization. After synchronization, cells were treated with DMSO or 0.2 µM MNNG and nuclear extracts were collected at the time points indicated. Endogenous MLH1 was immunoprecipitated with anti-MLH1 beads and immunoblotted for endogenous SHPRH and MLH1. Input was probed for SHPRH, MLH1, and Lamin A/C as the loading control. SHPRH-MLH1 interaction was seen at the 6-h timepoint, which correlates with the S phase in part **(A)**.

To further investigate the interaction between MLH1 and SHPRH, we looked at whether there was a cell-cycle dependency to the interaction, based on the data that the interaction is enhanced with MNNG-induced DNA damage. We first synchronized HeLa cells with a double thymidine block and followed cell cycle progression through DNA distribution by propidium idodide staining and fluorescence activated cell sorting (FACS) analysis. We carried out this experiment in the presence or absence of MNNG. After release from the double thymidine block, we observed the untreated and MNNG treated cells were beginning to move from G1 phase to S phase at 4 h and primarily in S phase by 6 h (Figure 6A). At the 10-h time point cells were in G2/M phase, and completed a cell cycle by 12 h (Figure 6A). Consistent with literature, we observed that MNNG induced a prolonged G2/M arrest occurring in the second cell cycle after treatment (24- and 36-h timepoints, Figure 6A) that is not observed in DMSO treated cells.

We then synchronized HeLa cells with a double thymidine block and collected nuclear lysates at the indicated time points corresponding with the cell cycle analysis above. The interaction between MLH1 and SHPRH is only observed by co-immunoprecipitation in S phase (6-h time point, Figure 6B), and is not detectable during G1 or G2/M.

### 3.7 Loss of SHRPH Leads to DNA Damage Resistance but Not Increased Mutation Rate

**FIGURE 7:**
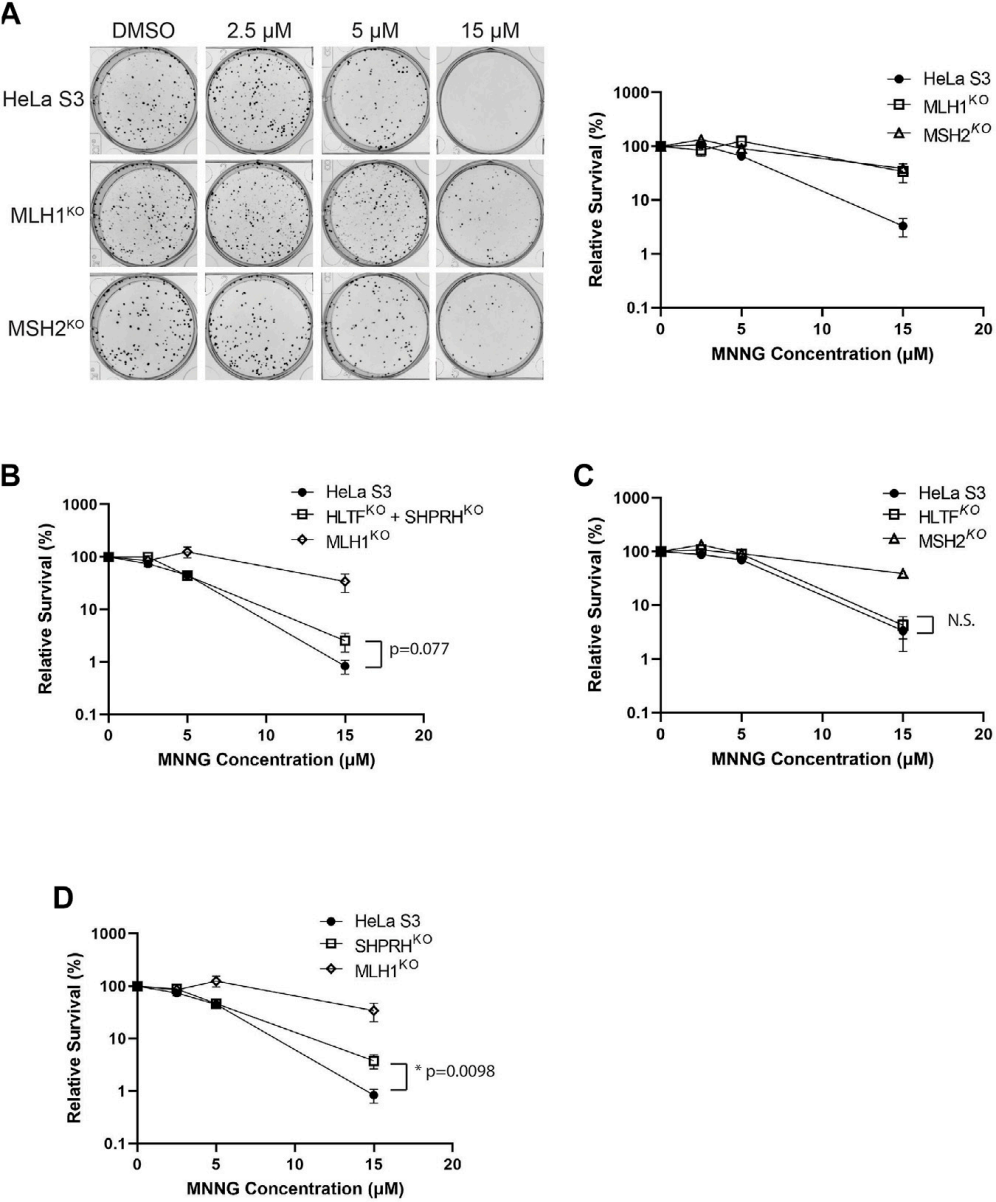
Loss of SHPRH results in resistance to alkylating agents. **(A)** HeLa S3 and CRISPR MLH1 and MSH2 knockout cells were seeded into 6-well plates for 24 h followed by a 2-h pre-treatment with O^6^-benzylguanine and a 1-h treatment of MNNG with O^6^-benzylguanine and seeded at a low density into a 6-well plate for a clonogenic survival assay. Left panel is a representative of stained colonies. Right panel is cell viability with colony counting. Data is shown as the mean of N = 3 with 4 replicate wells each ± SEM. **(B)** HeLa S3 and CRISPR HLTF + SHPRH double knockout cells were treated the same as part A for the clonogenic survival assay. Data is shown as the mean of N = 3 with 4 replicate wells ± SEM. Survival is compared to HeLa MLH1 KO survival from part **(A)**. **(C)** HeLa S3 and CRISPR HLTF knockout cells were treated the same as part A for the clonogenic survival assay. Data is shown as the mean of N = 3 with four replicate wells ± SEM. Survival is compared to HeLa MSH2 KO survival from part **(A)**. **(D)** HeLa S3 and CRISPR SHPRH knockout cells were treated the same as part A for the clonogenic survival assay. Data is shown as the mean of N = 3 with 4 replicate wells ± SEM. Survival is compared to HeLa MLH1 KO survival from part **(A)**. Statistical significance was determined by unpaired *t*-test *p < 0.05.

Treatment of mammalian cells with alkylating agents is known to cause MMR-mediated apoptosis in which loss of MMR activity causes increased alkylating agent resistance (Fu et al., 2012; Li et al., 2016). Given the interactions of HLTF and SHPRH with MMR proteins, we tested if loss of HLTF and/or SHPRH would similarly give rise to increased resistance to alkylation damage. To test this, we generated HeLa S3 cells in which either *MSH2*, *MLH1*, *SHPRH*, or *HLTF* was knocked out by CRISPR-Cas9. We also generated a cell line with both *SHPRH* and *HLTF* knocked out. Expression of the target proteins were totally eliminated in each cell line respectively and remained stably lost after greater than six passages (Supplementary Figure S1). MSH2 and MLH1 knockout cells show resistance to MNNG as previously reported for MMR deficient cells (Meikrantz et al., 1998; Fu et al., 2012) (Figure 7A). The HLTF and SHPRH double knock out cells showed a mild resistance to MNNG compared to the parental cells, although this did not reach the level of resistance equivalent to that of a total loss of MMR (Figure 7B). To determine if this phenotype was associated with a single Rad5 homolog or if it required loss of both proteins, we compared the MNNG sensitivity of the single knockout cell lines. HLTF knockout cells remained sensitive to MNNG in the clonogenic survival assay (Figure 7C); however, the SHPRH single knockout cell line showed moderate resistance to MNNG similar to that of the double knock out cell line (Figure 7D). While the resistance to MNNG was observed consistently with SHPRH loss, the cells were still markedly more sensitive to alkylating agents than cells that have totally lost MMR. Similar patterns of sensitivity to MNNG were observed for SHPRH and HLTF in a separate cell line that also has proficient MMR (HEK293) utilizing siRNA knock down of SHPRH, HLTF, or both as measured in a short-term survival MTS assay (Supplementary Figure S2) or long-term clonogenic assay (Supplementary Figure S3). This suggests that SHPRH may play a role in the promotion of apoptosis in a subset of alkylation-induced mispairs. This also demonstrates a functional difference between the two human Rad5 homologs in regards to MMR response to alkylating damage, potentially mediated by the evolutionary split of binding partners between the two homologs (Figure 10).

**FIGURE 8:**
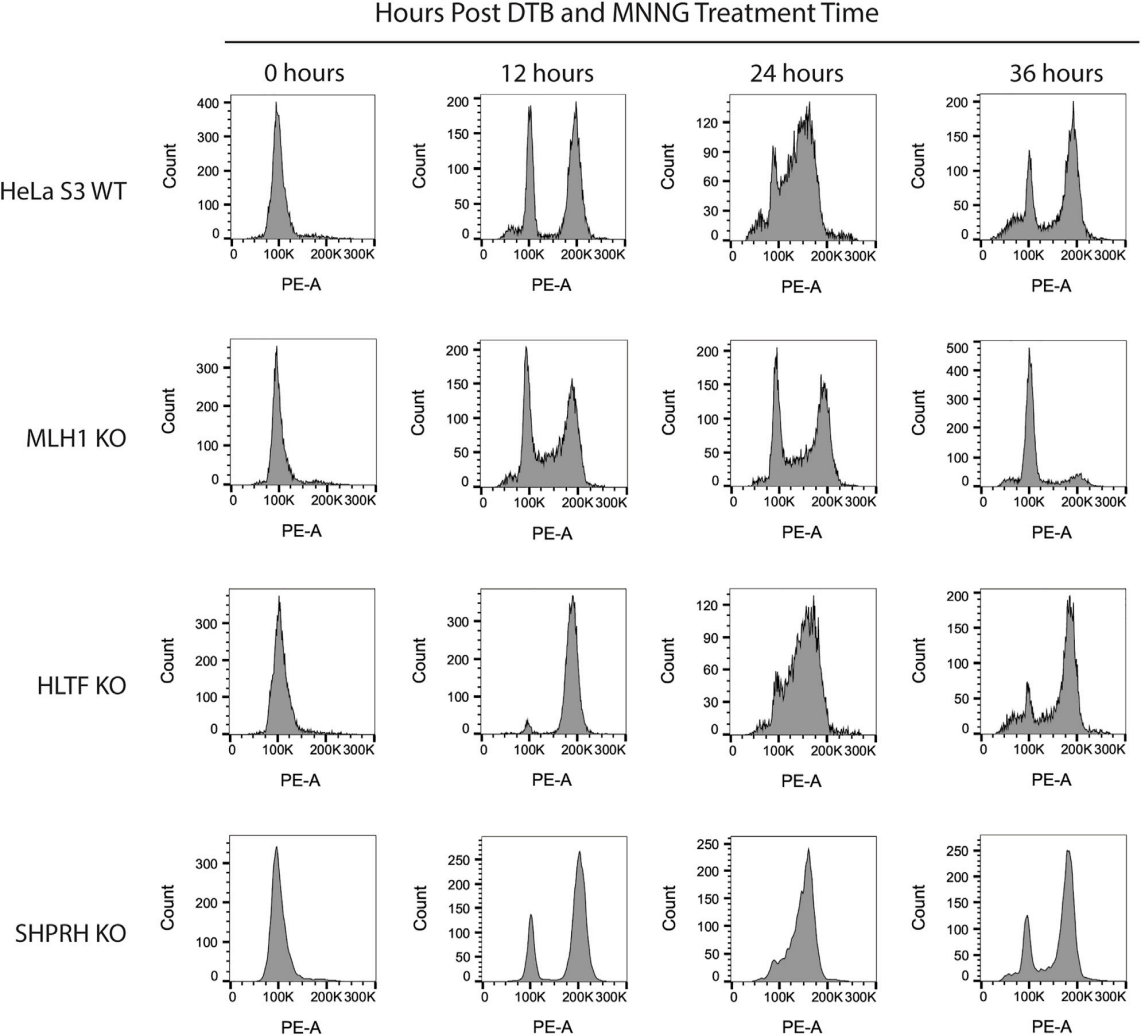
HLTF and SHPRH knock out cells retain MNNG induced G2/M arrest in the second cell cycle after damage. Cell cycle FACS analysis of HeLa WT, MLH1 KO, HLTF KO, and SHPRH KO cells treated with 0.2 µM MNNG for the times indicated after DTB synchronization. HeLa WT cells have G2/M arrest after the second cell cycle (24 h). HeLa MLH1 KO cells do not have the G2/M arrest that HeLa WT cells showed. HeLa HLTF KO and SHPRH KO both have G2/M arrest similar to the HeLa WT cells.

**FIGURE 9:**
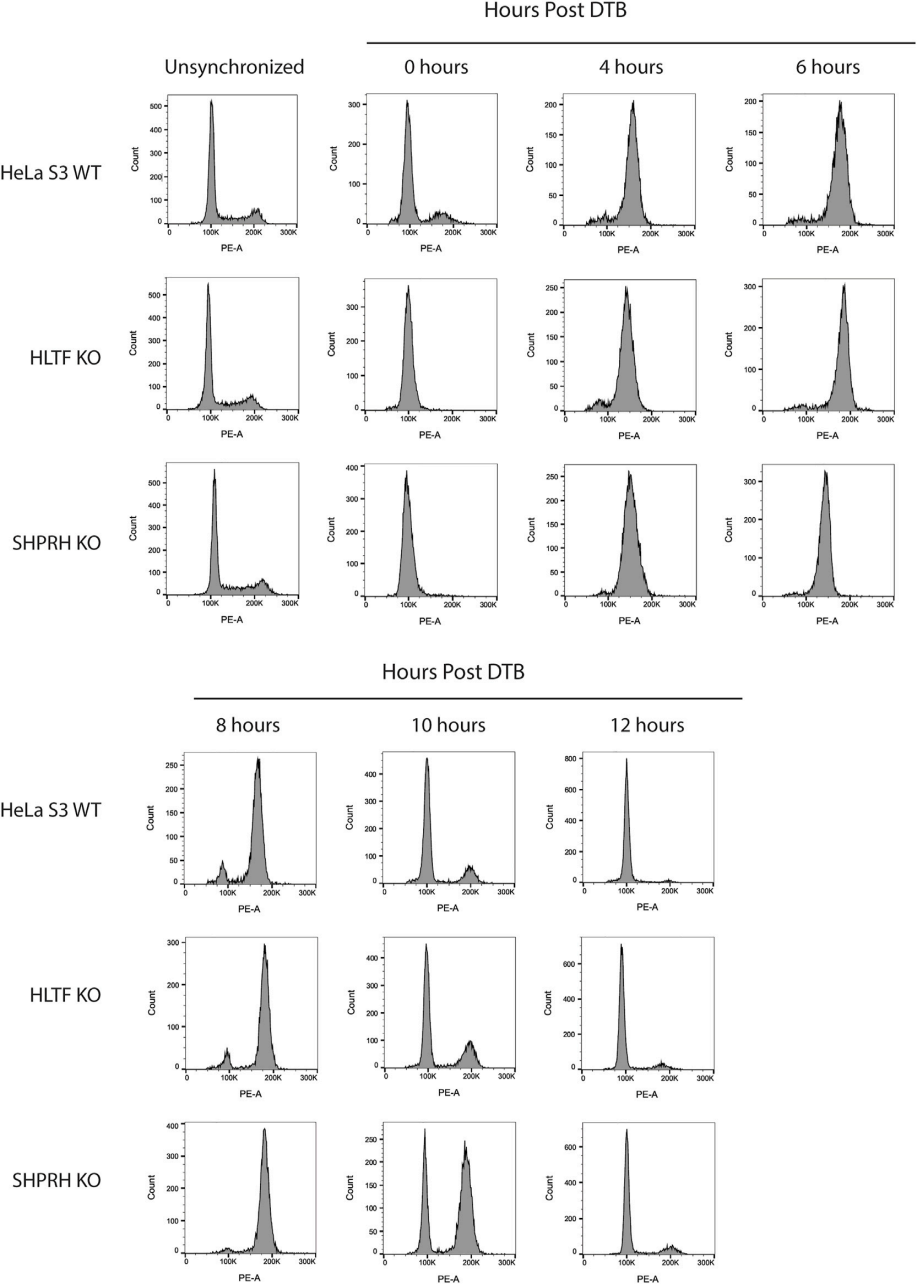
SHPRH knock out cells demonstrate delayed cell cycle without exogenous damage. Cell cycle FACS analysis of HeLa WT, HLTF KO, and SHPRH KO cells after release from DTB synchronization. HeLa HLTF KO cells follow the same cell cycle progression as the HeLa WT cells. HeLa SHPRH KO cells have a slower cell cycle progression and G2/M arrest compared to the HeLa WT cells.

To begin to determine the mechanisms of SHPRH involvement with the DNA MMR apoptotic response after alkylating damage, we also investigated the G2/M arrest occurring during the second cell cycle after exposure. A prolonged G2/M arrest in the second cell cycle after alkylation damage is well established phenotype for MMR-promoted apoptosis (Fu et al., 2012). Cells without MMR do not arrest or undergo apoptosis. We synchronized parental HeLa S3 cells and knock out cell lines using a double thymidine block and released after MNNG treatment. The parental cells showed the typical G2/M arrest starting at 24 h after treatment and maintained it through 48 h (Figure 8). The MLH1 knockout cells progressed through two normal cell cycles as reported in the literature (Figure 8). The HLTF knockout cells retained the G2/M arrest, consistent with their normal sensitivity to MNNG. Interestingly, the SHPRH knockout cells also retained a normal G2/M arrest despite a decreased sensitivity to MNNG (Figure 9). This suggests that SHPRH may play a role in the steps between G2/M arrest and the lack of resolution of the arrest that then leads to apoptosis. We also observed that untreated SHPRH knock out cells progressed through the cell cycle at a slower rate after synchronization, and that without damage they had a level of G2/M arrest (between 8 and 10 h, Figure 9). This was not observed in the HLTF knockout or parental HeLa cell lines (Figure 9). This change in cell cycle may be indicative of trouble resolving endogenous damage occurring in culture, potentially related to SHPRH’s role in translesion synthesis or template switching pathways.

Given the role of SHPRH in MMR-dependent apoptosis after alkylation damage, we wanted to determine if SHPRH, unlike Rad5 in *S. cerevisiae*, acted in the canonical MMR mutation avoidance pathway. To test this in our HeLa S3 knockout cells, we used the hypoxanthine phosphoribosyl transferase (HPRT) forward mutagenesis assay, as reported by Li et al. (Li et al., 2013). The parental HeLa S3 cells had a mutation frequency less than 4.78 × 10^−6^ and the MLH1 and MSH2 knockout cells had increased mutation frequency of about 2.45 × 10^−4^, similar to the reported frequency for other MMR deficient cell lines (Table 3 (Li et al., 2013)). The SHPRH knockout cells had an estimated rate about equal to the parental cell lines, without any significant colony formation observed at even at higher plating densities (Table 3). Together this data suggests that SHPRH influences the MMR mediated response to alkylation-induced mispairs, but not repair of replication errors through canonical MMR.

**TABLE 3:** HPRT mutation frequency.

Cell line	*HPRT* Mutation frequency
HeLa S3	<4.78 × 10^−6^
SHPRH KO	<9.31 × 10^−6^
MLH1 KO	2.44 [2.29–2.70] x10^−4^
MSH2 KO	2.47 [1.82–3.08] x10^−4^

Reported frequency is the median frequency with 95% confidence interval in square brackets calculated as described in materials and methods. n = 6 per cell line.

## 4 Discussion

The identification of the MIP box Mlh1-binding motif (Dherin et al., 2009) and, more recently, the SHIP box Msh2-binding motif (Goellner et al., 2018) have revealed how many proteins are recruited to sites of MMR. These proteins include those directly involved in MMR (*e.g.* Exo1) and have identified a number of other proteins whose roles in MMR and MMR-mediated processes are less well understood, including *S. cerevisiae* Ntg2, Sgs1, Fun30, and Dpb3 and human FAN1, SMARCAD1, WDHD1, and MCM9 (Dherin et al., 2009; Traver et al., 2015; Chen et al., 2016; Goellner et al., 2018; Terui et al., 2018; Takeishi et al., 2020; Goold et al., 2021; Porro et al., 2021). Here, we have used analysis of candidate MIP and SHIP box sequences to identify *S. cerevisiae* Rad5 as a MIP box-mediated Mlh1 interactor and a SHIP box-independent Msh2 interactor. These interactions are conserved through evolution to the human homologs of Rad5, HLTF, and SHPRH; however, the interaction seems to have split during evolution between the two homologs, with HLTF retaining MSH2 binding and SHPRH retaining MLH1 binding.

Why Rad5 homologs can bind to MMR proteins remains an open question. Numerous screens for mutations that cause MMR defects in *S. cerevisiae* have not identified *rad5* mutations (Huang et al., 2003; Schmidt et al., 2017). Unlike forward mutation assays like the Can1^R^ and HPRT assays, *hom3-10* and similar frameshift reversion assays measure mutation events that are primarily specific to MMR defects (Marsischky et al., 1996; Harfe and Jinks-Robertson, 1999). Sequence analysis of the mutation spectra in MMR-deficient strains has shown that the primary *hom3-10*-reverting mutation is T7 →T6 (100%, 73 of 73 in MMR-defective genotypes; and 93%, 162 of 181 in partial MMR-defective genotypes) (Tishkoff et al., 1997; Flores-Rozas and Kolodner, 1998; Calil et al., 2021). The *rad5Δ* mutation caused only a very small increase in the *hom3-10* frameshift reversion rate, and this rate increase is attributable to a different spectrum of mutations than those expected due to an MMR defect (39% T7 →T6 frameshifts). These results suggest that Rad5 either does not play a major role in mutation avoidance by MMR, consistent with prior results (Johnson et al., 1992), or it is redundant with other MMR subpathways, similar to other MMR components such as Exo1 (Goellner et al., 2015).

To model the MMR-mediated response to S_N_1-type alkylating agents in budding yeast, studies must be carried out in strains that have a *rad52Δmgt1Δ* double mutation background to overcome immediate repair by either direct reversal or homologous recombination pathways that are highly efficient in yeast (Cejka et al., 2005). The sensitivity of *rad5Δ* strains to replication blocking lesions specific to S_N_2-type alkylating agents, such as MMS, has been heavily studied in the context of PRR (Xu et al., 2016). However, to our knowledge, few studies have looked at *rad5Δ* mutation containing strains in the context of SN1-type agents in the appropriate background to determine their impact on non-canonical MMR. Cjeka et al. did conduct a genome wide screen using the yeast deletion library in the *rad52Δmgt1Δ* genetic background and did not identify any factors beyond MMR as having a significant loss of sensitivity to MNNG (Cejka and Jiricny, 2008). However, in the same manuscript they did a second screen only in the presence of *mgt1Δ* to identify factors that may help resolve MMR mediated toxic intermediates. In this second screen *RAD5* was identified and interestingly was far more sensitive than other members of the PRR pathway (Cejka and Jiricny, 2008), suggesting that *RAD5* may be playing a unique role that we hypothesize is due to its physical interactions with MMR.

Based on these results, we have focused our efforts on understanding the role of Rad5 human homologs, HLTF and SHPRH, in non-canonical functions of MMR. Since our report that Msh2 interacts with the Fun30 helicase (SMARCAD1 in humans), another group has confirmed the human MSH2-SMARCAD1 interaction and demonstrated that SMARCAD1 KO cell lines are moderately resistant to alkylating agent-induced apoptosis likely through changes in the chromatin association of MMR proteins (Takeishi et al., 2020). Similarly, we found that depletion or knockout of SHPRH results in moderate resistance to alkylation-induced cell death, consistent with the Rad5-Mlh1 MIP box interaction and the SHPRH-MLH1 interaction. Interestingly, the SHPRH knockout lines retain the MNNG-mediated G2/M arrest but have reduced cell death. SHPRH has several functional domains, including a helicase and E3 ligase domain (Elserafy et al., 2018). Complementation studies are ongoing to determine which SHPRH domains are critical to mediating sensitivity to alkylation damage. Intriguingly, both SHPRH and SMARCAD1 are SNF2-family DNA helicases albeit with very different functions: fork reversal and nucleosome remodeling, respectively. It is currently unclear if there is any redundancy or additive effect between SHPRH and SMARCAD1 roles in influencing this pathway.

**FIGURE 10:**
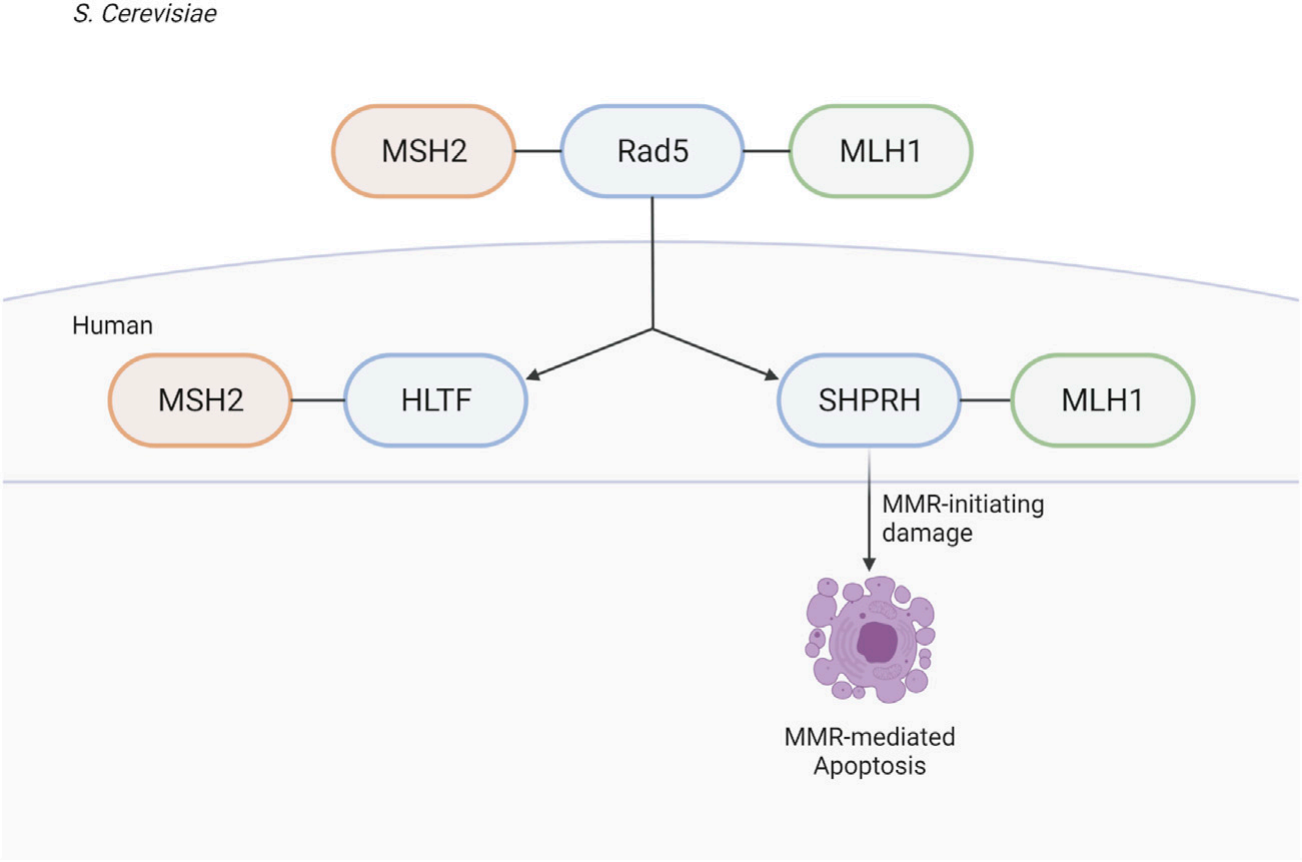
Rad5 and human homologs interact with the MMR pathway. *Saccharomyces cerevisiae* helicase/E3 ligase Rad5 interacts with both key players in eukaryotic MMR, Msh2 and Mlh1. Rad5 has two human homologs, HLTF and SHPRH. Binding to the MMR pathway is conserved throughout evolution, but split between the two human homologs with HLTF binding MSH2 and SHPRH binding MLH1. SHPRH plays a role in apoptosis after alkylation damage as depletion of SHPRH results in mild resistance to MNNG. Created with BioRender.com.

We find it especially interesting that while the interactions of both Msh2 and Mlh1 with Rad5 are conserved through evolution to the human homologs, that the binding sites seem to have been split between the two homologs (Figure 10). Given the differences of HLTF and SHPRH in alkylation sensitivity, it seems possible that the Msh2-Rad5 and Mlh1-Rad5 interactions have different functional roles that are retained in different Rad5 homologs after gene duplication and specialization (Ohno, 1970). An intriguing possibility, since Rad5 does not appear to act in the canonical MMR mutation avoidance pathway, is that the HLTF-MSH2 interaction acts in a separate non-canonical role of MMR such as heteroduplex rejection (Tham et al., 2016) or that MSH2 influences the role of HLTF in PRR. Several groups have shown an interaction between nuclease FAN1 and MLH1, mediated by a MIP box and an additional MLH1-interaction domain (Goold et al., 2021; Porro et al., 2021). This binding seems to influence apoptotic response to MNU and also control FAN1’s role in trinucleotide repeat stabilization and interstrand cross-link repair (Rikitake et al., 2020; Porro et al., 2021). HLTF and SHPRH may be similarly impacted by MMR interactions that affect their previously identified cellular roles. Porro et al. also demonstrate that phosphorylation of the MIP box changes the association between FAN1 and MLH1, raising questions on whether the interactions between MMR proteins and Rad5 homologs may also be regulated by post-translational events.

## Data Availability

The original contributions presented in the study are included in the article/Supplementary Material, further inquiries can be directed to the corresponding author.

## Supplementary Material

Supplementary Figure S1 Generation of knockout cells by CRISPR-Cas9. **(A)** Schematic of sgRNA sequence and target for generation of MSH2 knock out Hela S3 cells (top). Immunoblot of MSH2 protein levels in parental cells and selected clones after 6 continuous passages. MSH2 knockout was retained in clone 2 but re-expressed in clone 4. **(B)** Schematic of sgRNA sequence and target for generation of HLTF knock out Hela S3 cells (top). Immunoblot of HLTF protein levels in parental cells and selected clones after 6 continuous passages. HLTF knockout was retained in clone 3 and clone 4. The double knockout cell line was made by knockout of SHPRH in the HLTF knockout background. **(C)** Schematic of sgRNA sequence and target for generation of MLH1 knock out Hela S3 cells (top). Immunoblot of MLH1 protein levels in parental cells and selected clones after 6 continuous passages. MLH1 knockout was retained in clone 3 and clone 4. **(D)** Schematic of sgRNA sequence and target for generation of SHPRH knock out Hela S3 cells (top). Immunoblot of SHPRH protein levels in parental cells and selected clones after 6 continuous passages. SHPRH knockout was retained in clone B2 and clone C3.

Supplementary Figure S2 Loss of SHPRH results in resistance to alkylating agents. **(A)** HEK293 cells were co-transfected with siRNA to both HLTF and SHPRH. Cells were seeded into 96-well plates for 24 h followed by a 1-h treatment with MNNG and assayed for survival after 72 h by MTS assay. Data is shown as the mean of *N* = 3 ± SEM. Efficiency of knockdown for the used siRNA duplex is shown in the right panel. **(B)** HEK293 cells were transfected with siRNA to HLTF. Cells were seeded into 96-well plates for 24 h followed by a 1-h treatment with MNNG and assayed for survival after 72 h by MTS assay. Data is shown as the mean of *N* = 3 ± SEM. Efficiency of knockdown for the used siRNA duplex is shown in the right panel. **(C)** HEK293 cells were transfected with siRNA to SHPRH. Cells were seeded into 96-well plates for 24 h followed by a 1-h treatment with MNNG and assayed for survival after 72 h by MTS assay. Data is shown as the mean of *N* = 3 ± SEM. Efficiency of knockdown for the used siRNA duplex is shown in the right panel.

Supplementary Figure S3 Clonogenic survival assay of HEK293 cells transfected with siSHPRH. HEK293 cells with SHPRH knocked down with siRNA were treated with indicated doses of the alkylating agent MNNG in a long-term clonogenic survival assay. HEK293 cells do not form countable colonies. Visually, siSHPRH cells have greater survival than siScrambled cells.
