# Supplementary figures and images for: Rad5 and Its Human Homologs, HLTF and SHPRH, Are Novel Interactors of Mismatch Repair
Source: Front Cell Dev Biol. 2022 Jun 16;10:843121. doi: 10.3389/fcell.2022.843121 (PMC9243396; doi:10.3389/fcell.2022.843121)

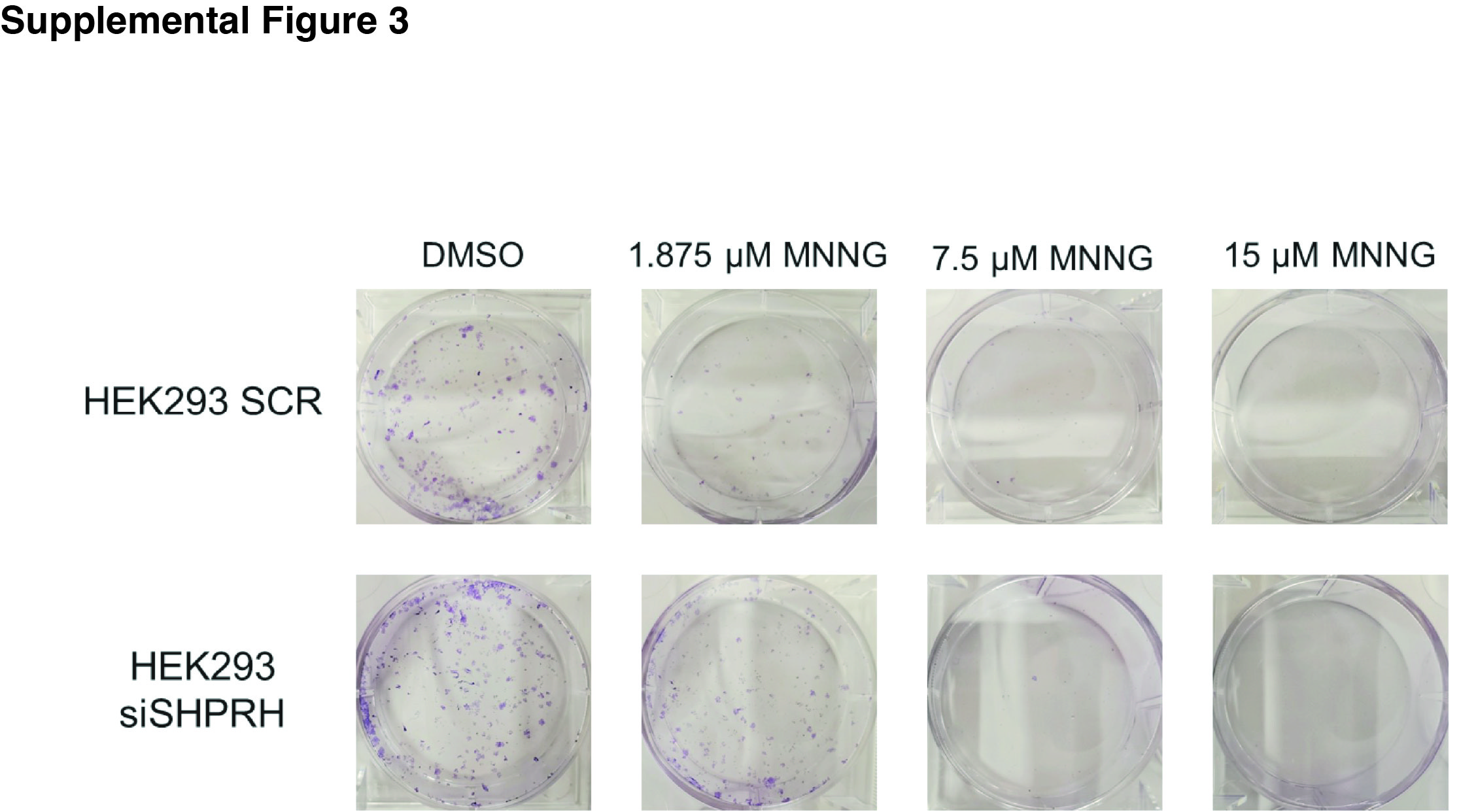

Supplement: Supplementary file 1 [file Image3.JPEG]

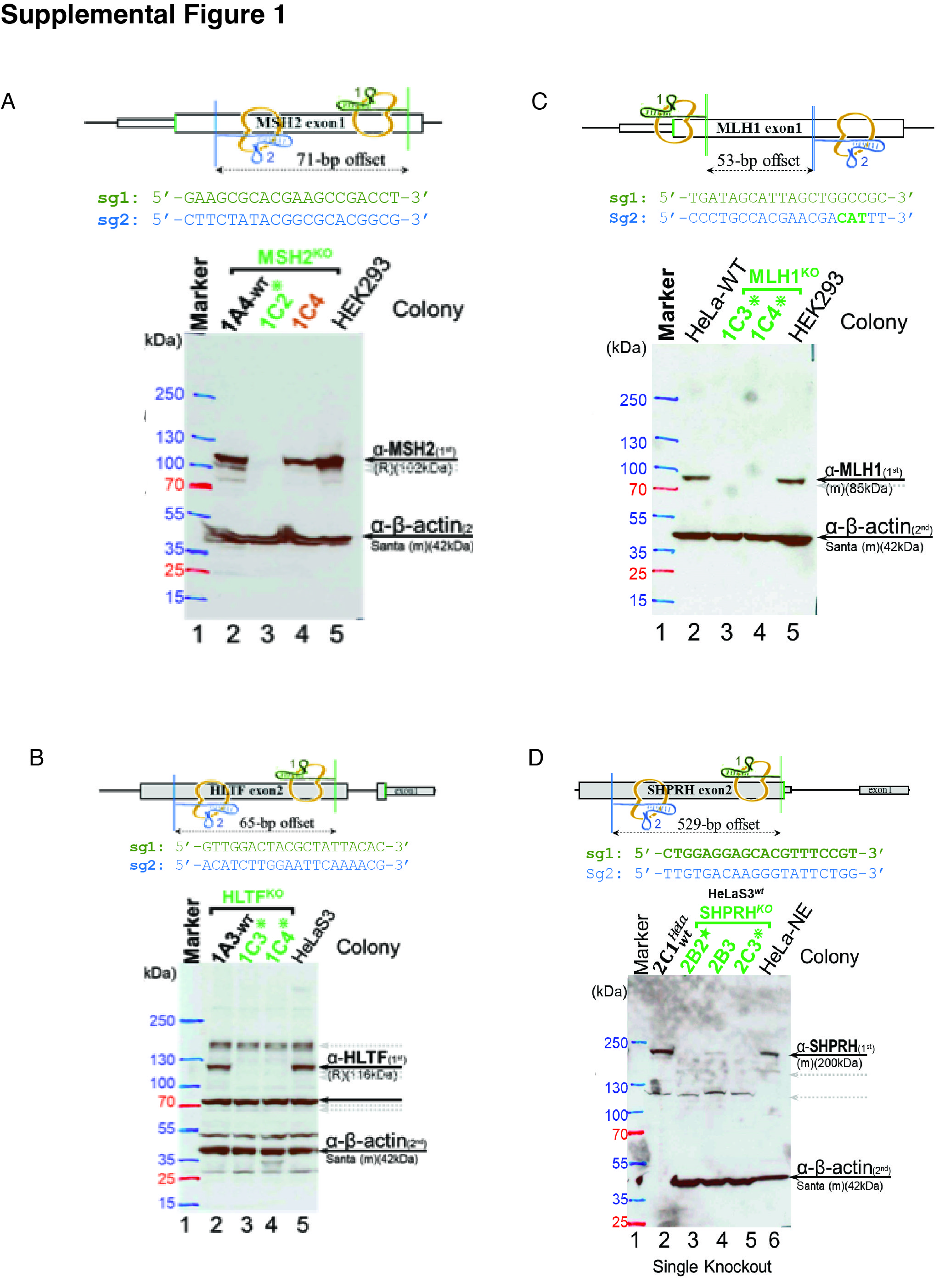

Supplement: Supplementary file 3 [file Image1.JPEG]

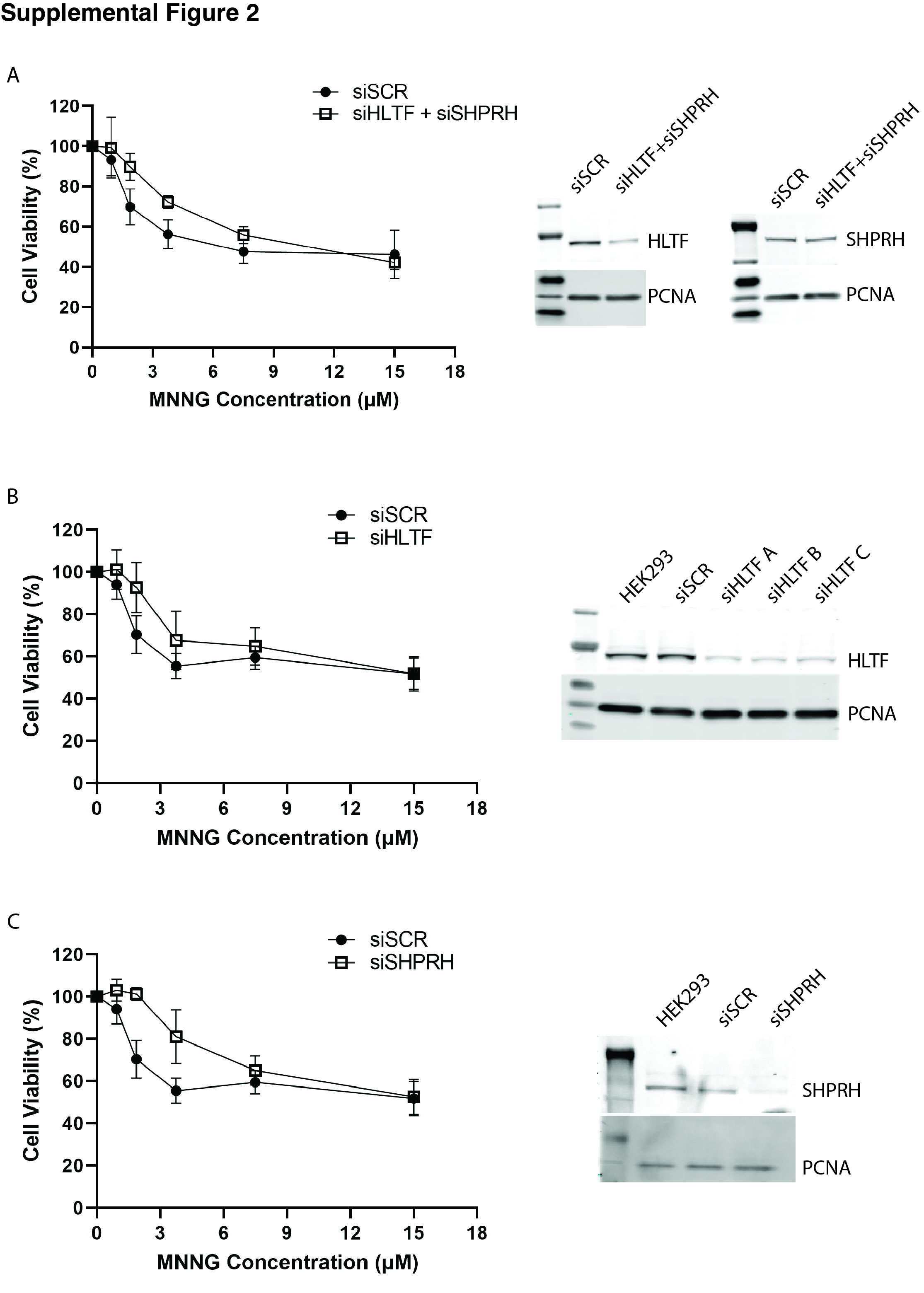

Supplement: Supplementary file 4 [file Image2.JPEG]
