# Supplementary material for: Rad5 and Its Human Homologs, HLTF and SHPRH, Are Novel Interactors of Mismatch Repair
Source: Front Cell Dev Biol. 2022 Jun 16;10:843121. doi: 10.3389/fcell.2022.843121 (PMC9243396; doi:10.3389/fcell.2022.843121)
Supplement: Supplementary file 2 [file Table1.DOCX]

**Supplemental Figure Legends**

**Supplemental Figure 1. Generation of knockout cells by CRISPR-Cas9**

A. Schematic of sgRNA sequence and target for generation of Msh2 knock out Hela S3 cells (top). Immunoblot of Msh2 protein levels in parental cells and selected clones after 6 continuous passages. Msh2 knockout was retained in clone 2 but reexpressed in clone 4. B. Schematic of sgRNA sequence and target for generation of HLTF knock out Hela S3 cells (top). Immunoblot of HLTF protein levels in parental cells and selected clones after 6 continuous passages. HLTF knockout was retained in clone 3 and clone 4. The double knock out cell line was made by knock out of SHPRH in the HLTF knock out background C. Schematic of sgRNA sequence and target for generation of MLH1 knock out Hela S3 cells (top). Immunoblot of MLH1 protein levels in parental cells and selected clones after 6 continuous passages. MLH1 knockout was retained in clone 3 and clone 4. D. Schematic of sgRNA sequence and target for generation of SHPRH knock out Hela S3 cells (top). Immunoblot of SHPRH protein levels in parental cells and selected clones after 6 continuous passages. SHPRH knockout was retained in clone B2 and clone C3.

**Supplemental Figure 2. Loss of SHPRH results in resistance to alkylating agents**

**A.** HEK293 cells were co-transfected with siRNA to both HLTF and SHPRH. Cells were seeded into 96 well plates for 24 hours followed by treatment with a 1-hour treatment of MNNG and assayed for survival after 72 hours by MTS assay. Data is shown as the mean of N=3 +/- SEM. Efficiency of knockdown for the used siRNA duplex is shown in the right panel. **B.** HEK293 cells were transfected with siRNA to HLTF. Cells were seeded into 96 well plates for 24 hours followed by treatment with a 1-hour treatment of MNNG and assayed for survival after 72 hours by MTS assay. Data is shown as the mean of N=3 +/- SEM. Efficiency of knockdown for the used siRNA duplex is shown in the right panel. **C.** HEK293 cells were transfected with siRNA to SHPRH. Cells were seeded into 96 well plates for 24 hours followed by treatment with a 1-hour treatment of MNNG and assayed for survival after 72 hours by MTS assay. Data is shown as the mean of N=3 +/- SEM. Efficiency of knockdown for the used siRNA duplex is shown in the right panel.

**Supplemental Figure 3. Clonogenic survival assay of HEK293 cells transfected with siSHPRH**

HEK293 cells with SHPRH knocked down with siRNA were treated with indicated doses of alkylating agents in a long-term clonogenic survival assay. HEK293 cells do not form countable colonies. Visually, siSHPRH cells have greater survival than siScrambled cells.
